# IL‐6 signaling regulates the inflammatory response without impacting pathogen burden during influenza‐associated pulmonary aspergillosis

**DOI:** 10.14814/phy2.70372

**Published:** 2025-05-27

**Authors:** Lokesh Sharma, Ravineel B. Singh, Nathaniel J. Tolman, Caden Ngeow, Alexis M. Duray, Nima Naghshtabrizi, Aijaz Ahmad, William Bain, Keven M. Robinson

**Affiliations:** ^1^ Division of Pulmonary, Allergy, Critical Care and Sleep Medicine, Department of Medicine University of Pittsburgh Pittsburgh Pennsylvania USA; ^2^ Division of Pulmonary Medicine, Department of Pediatrics University of Pittsburgh Pittsburgh Pennsylvania USA

**Keywords:** aspergillus, IL‐6 signaling, inflammation, influenza, influenza associated pulmonary aspergillosis

## Abstract

Viral infections increase host susceptibility to opportunistic pathogens like Aspergillus fumigatus (AF), exacerbating disease severity and prolonging its clinical course. Interleukin‐6 (IL‐6) drives pathological inflammation in viral infections such as COVID‐19, but its role in influenza, particularly with secondary AF infection, remains unclear. Using a mouse model of post‐influenza AF infection, including IL‐6 knockout mice, we found that IL‐6 signaling promotes neutrophilic lung inflammation but is not required for pathogen clearance of either influenza or AF. However, IL‐6 deficiency increases epithelial cell damage, as indicated by elevated RAGE levels in bronchoalveolar lavage fluid. In contrast, lung capillary permeability (measured by IgM levels in BAL) and tissue injury (assessed histologically) remain unaffected in the absence of IL‐6 signaling. These findings reveal a nuanced role for IL‐6 in post‐influenza AF infection, underscoring its contribution to lung inflammation and epithelial integrity.

## 1 INTRODUCTION

Both Influenza A and SARS‐CoV‐2 are highly transmittable respiratory viruses that cause seasonal epidemics and pandemics. Interleukin‐6 (IL‐6) is upregulated during both influenza and Covid‐19 infection and has been linked to disease severity (Hafez et al., 2023; Hagau et al., 2010; Huang et al., 2020; Kaiser et al., 2001; La Gruta et al., 2007; Liu et al., 2020; Zhang et al., 2020). Immunomodulatory therapies, including monoclonal antibodies targeting the IL‐6 receptor or cytokine‐receptor binding affinity, have been shown to improve outcomes during SARS‐CoV‐2 infection (Rosas et al., 2021; Salama et al., 2021). However, IL‐6 has been shown to be protective during influenza infection, with IL‐6 deficient mice having increased morbidity and mortality (Dienz et al., 2012; Yang et al., 2017). A key clinical question that remains unanswered is if immunomodulatory therapies used during SARS‐CoV‐2 will also be beneficial during influenza infection.

*Aspergillus fumigatus* is an environmental fungus that can cause severe and life threatening infections in immunocompromised hosts (Ben‐Ami et al., 2010; Latgé & Chamilos, 2019). The post‐viral lung environment renders the host susceptible to a wide range of secondary infections, including those caused by *A. fumigatus* (Feys et al., 2024; Rothberg et al., 2008; Tobin et al., 2020). During the COVID‐19 pandemic, COVID‐19 associated pulmonary aspergillosis (CAPA) emerged as an important complication of SARS‐CoV‐2 infection (Arastehfar et al., 2020). Severe influenza infection is also an established risk factor for the development of influenza‐associated invasive pulmonary aspergillosis (IAPA) in both immunocompetent and immunocompromised individuals. IAPA causes significant morbidity and mortality in affected patients compared to those with singular influenza infection (Van De Veerdonk et al., 2017). The role of IL‐6 during IAPA remains unclear and increased understanding is critical prior to the use of immunomodulatory therapies targeting IL‐6 during influenza infection. In this study, we used a murine model of IAPA to determine if IL‐6 contributes to host susceptibility to IAPA. IL‐6 modulatory therapies such as tocilizumab are ineffective in mouse models (Lokau, Kleinegger, et al., 2020; Lokau, Waetzig, et al., 2020; Okazaki et al., 2002), therefore, we utilized IL‐6 deficient mice to model IL‐6 inhibition during influenza respiratory infection.

## 2 RESULTS

### 2.1 Decreased airway neutrophilia in IL‐6 deficient mice during IAPA

**FIGURE 1:**
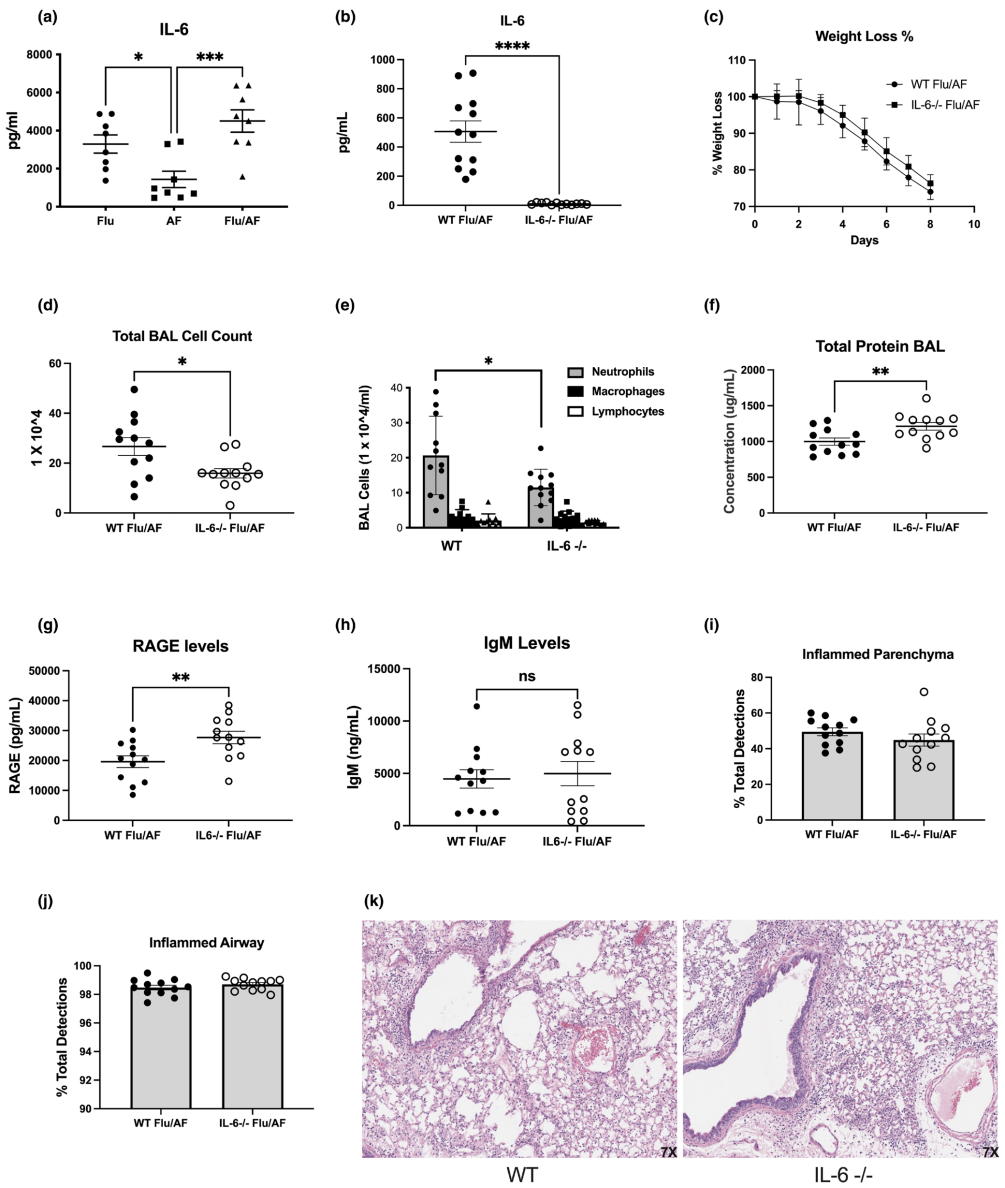
Decreased airway neutrophilia in IL‐6 deficient mice during IAPA. C57BL/6 mice (wild‐type) were infected with influenza A H1N1 PR/8/34 on day 0 and subsequently challenged with 2.5 × 10^7^ *Aspergillus fumigatus* (AF) at 6 days post‐infection. Lung samples were collected at 48 h post‐AF challenge (*n* = 8). (a) IL‐6 concentrations in lung homogenate measured by 23‐plex immunoassay. C57BL/6 mice (wild‐type) and IL‐6−/− were serially infected with influenza A and AF as described and samples were collected at 48 h post‐AF challenge (*n* = 12). (b) IL‐6 concentrations in lung homogenate measured by 23‐plex immunoassay. (c) Weight loss measured through Day 8 post‐infection. (d) BAL fluid total cell counts. (e) BAL differential cell counts. (f) Total protein in BAL fluid. (g) RAGE levels in BAL fluid measured by ELISA. (h) IgM levels in BAL fluid measured by ELISA. (i–k) Representative histology of H&E‐stained left lung sections and scoring of parenchymal and airway inflammation. Data were compiled from two independent experiments and data points reflect individual values ± SEM with the exception of continuous data in panel (c) that reflects median value ± SD. Significance was tested by the unpaired *t* test (for 2 means) after normality was confirmed with statistical significance marked as *p < 0.05, **p < 0.005, and ***p < 0.0005.

Influenza infection increases host susceptibility to secondary pulmonary infections, including invasive pulmonary aspergillosis. We have previously published a murine model of IAPA where C57BL/6 male mice were challenged with a sublethal dose of influenza A PR/8/34 H1N1 for 6 days, followed by *A. fumigatus* (ATCC strain 42202) conidia. Mice were euthanized 2 days post AF infection to assess pathogen burden, tissue injury, and inflammation (Tobin et al., 2020). We observed increased fungal burden, viral burden, lung inflammation, and mortality compared to mice singularly infected with aspergillus (Tobin et al., 2020). In our current studies, we utilized this model as it mimics clinical susceptibility to *A. fumigatus* seen in patients that are critically ill with influenza. First, we measured IL‐6 levels in mice infected with singular aspergillus, singular influenza, and dual infected with both influenza and aspergillus and observed increased levels of IL‐6 in both influenza‐infected and dual‐infected mice compared to mice infected with aspergillus alone (Figure 1a). IL‐6 inhibitors have been shown to be protective in patients with COVID‐19 (Liu et al., 2020; Zhang et al., 2020) and it has been proposed that IL‐6 inhibitors may also be protective during severe influenza infection (Hays et al., 2022). Immunomodulation during influenza infection may potentially affect the development of subsequent secondary pulmonary infections. Given IL‐6 is protective during invasive pulmonary aspergillosis and also shown to be increased in our murine model of IAPA compared to aspergillus alone (Figure 1a), we sought to examine how IL‐6 deletion affects IAPA (Cenci et al., 2001). C57BL/6 and IL‐6 KO male mice were challenged with a sublethal dose of influenza A PR/8/34 H1N1 (2000 PFU) for 6 d, followed by 2.5 × 10^7^ *A. fumigatus* (ATCC strain 42202) conidia, and after 48 h, tissues were harvested. Our data confirm that IL‐6 is present in the lung during IAPA, which was completely absent in IL‐6 knockout mice (Figure 1b). Although we did not observe any difference in weight loss in the absence of IL‐6 (Figure 1c), we found that IL‐6 deficiency significantly ameliorated inflammatory cells in the alveolar space (Figure 1d). Differential cell analysis identified decreased neutrophil accumulation as the major contributor to this reduced inflammatory response (Figure 1e). To investigate whether reduced neutrophil accumulation alters lung permeability, we measured the total protein content in the bronchoalveolar lavage fluid (BAL) (Figure 1f). Interestingly, we observed a significant increase in total protein content in the IL‐6 deficient mice, indicating either increased permeability or increased lung cell death in IL‐6 knockout mice. To identify the source of this elevated total protein in the BAL, we measured the levels of RAGE (marker of epithelial cell damage) and IgM (for lung permeability). Our data show that while IL‐6 knockout mice had a significant increase in the RAGE levels (1G), but not an increase in the BAL IgM levels (Figure 1h), suggesting a role for IL‐6 in maintaining epithelial viability during IAPA. We further investigated the role of IL‐6 signaling and lung injury through histologic analysis. Our unbiased approach revealed that the lack of IL‐6 did not alter the overall inflammatory pathology in lung tissue, including the parenchyma (Figure 1i) and airways (Figure 1j). Overall, the IL‐6 knockout mice exhibited similar tissue injury compared to wild‐type mice (Figure 1k). Our data show that IL‐6 deficiency inhibits neutrophilia during IAPA and increases lung epithelial cell damage; however, IL‐6 deficiency does not alter overall lung inflammation.

### 2.2 Preserved antifungal immunity in IL‐6 deficient mice during IAPA

**FIGURE 2:**
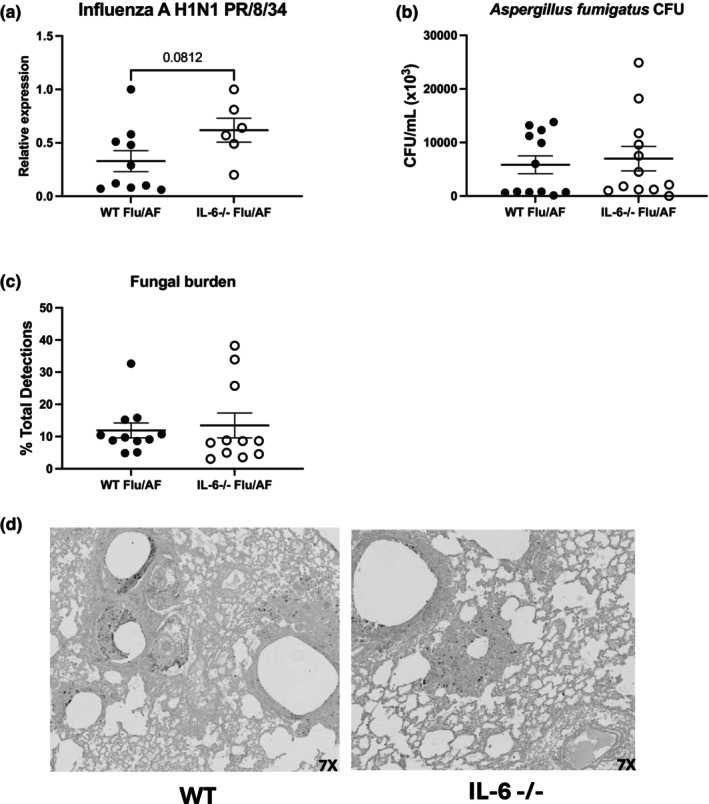
Preserved viral and fungal clearance in IL‐6 deficient mice during IAPA. C57BL/6 and IL‐6−/− mice were infected with influenza A H1N1 PR/8/34 on day 0 and subsequently challenged with 2.5 × 10^7^ *Aspergillus fumigatus* (AF) at 6 days post‐infection. Lung samples were collected at 48 h post‐AF challenge. (a) Influenza matrix protein expression measured in lung RNA (*n* = 6–12). (b) Lung fungal burden was measured by CFU (*n* = 12). (c, d) Representative histology of GMS‐stained left lung sections and lung fungal burden measured by GMS‐stained left lung sections (*n* = 11). Data were compiled from two independent experiments and data points reflect individual values ± SEM. Significance was tested by the unpaired *t*‐test (for 2 means) after normality was confirmed with statistical significance marked as *p < 0.05, **p < 0.005, and ***p < 0.0005.

To test whether this decreased neutrophil recruitment impairs the host ability to clear the invading pathogens, we investigated the burden of influenza and aspergillus during IAPA in IL‐6 knockout mice. We observed a trend towards increased influenza viral burden in IL‐6 deficient mice; however, the difference was not statistically significant (Figure 2a). Despite the trend towards increased influenza viral burden in IL‐6 deficient mice, we observed no differences in fungal burden. To investigate fungal burden, we measured colony forming units (CFU) from lung homogenate (Figure 2b) and also scored GMS‐stained lungs. GMS‐stained lungs were scored for the presence of conidia or hyphae (Figure 2c,d) and no differences were observed between IL‐6 knockout and wild‐type mice. Our data demonstrate a preserved antifungal immunity during IAPA despite perturbation of IL‐6 signaling.

### 2.3 IL‐6 deficiency alters cytokine and chemokine levels during IAPA

**FIGURE 3:**
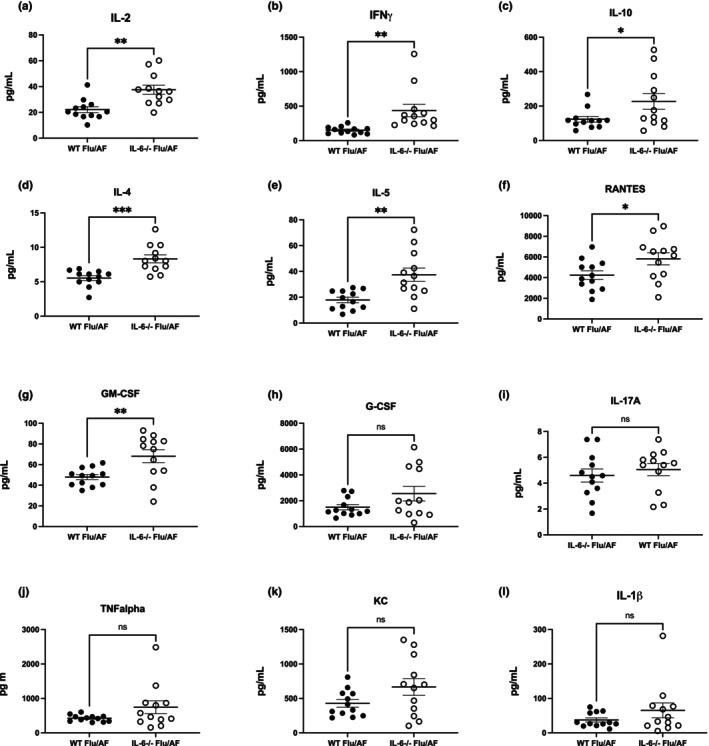
Enhanced type I and II immunity and chemokine levels during IAPA. C57BL/6 and IL‐6−/− mice were infected with influenza A H1N1 PR/8/34 on day 0 and subsequently challenged with 2.5 × 10^7^ *Aspergillus fumigatus* (AF) at 6 days post‐infection. Lung samples were collected at 48 h post‐AF challenge (*n* = 12). (a–l) Protein concentrations in lung homogenate measured by 23‐plex immunoassay. Data were compiled from two independent experiments and data points reflect individual values ± SEM. Significance was tested by the unpaired *t*‐test (for 2 means) after normality was confirmed with statistical significance marked as *p < 0.05, **p < 0.005, and ***p < 0.0005.

To gain mechanistic insight into the divergent phenotype of decreased neutrophilia but elevated epithelial injury (elevated RAGE levels) in the lung, we measured cytokine and chemokine levels in the lungs of infected mice. Our data show that the absence of IL‐6 led to a significant elevation in IL‐2, IFN‐γ, IL‐4, IL‐5, and IL‐10 (Figure 3a–e). Similarly, we observed an increase in chemokines RANTES and GM‐CSF during IAPA in IL‐6 knockout mice (Figure 3f,g). However, we did not observe changes in G‐CSF, IL‐17A, TNFα, KC, and IL‐1β (Figure 3h–l). Taken together, our data suggest that the deletion of IL‐6 enhances Type I and Type 2 responses and increases select chemokine responses in the lung during IAPA.

## 3 DISCUSSION

Inflammation is a key cellular response that is essential for host immunity against infection and to repair tissue injury that occurs during infection. However, inflammation can also promote tissue injury, which can be detrimental to host survival during infection. The role of inflammation is highly complicated during coinfections and poly‐microbial infections, as different types of infection regulate different inflammatory responses that can either enhance or oppose one another. Prior studies have thoroughly investigated IL‐6 signaling in both singular influenza and singular aspergillus infections in the lung and observed beneficial effects of IL‐6 in these models (Cenci et al., 2001; Dienz et al., 2012; Lauder et al., 2013; Yang et al., 2017). IL‐6 deficient mice infected with influenza have increased morbidity, mortality, and viral burden compared to wild‐type controls. IL‐6 receptor deficient mice also have increased mortality and increased immunopathology visualized on histologic lung sections compared to wild‐type controls (Dienz et al., 2012; Lauder et al., 2013). Cytokine, chemokine, and interferon levels are altered during influenza infection in IL‐6 deficient mice, including increased TGFβ by day 6 post‐infection and increased TNFα, IFNα, and CCL2 by day 7 post‐infection. However, no changes in IL‐1β or IFNγ have been observed in IL‐6 deficient mice compared to wild‐type controls (Dienz et al., 2012; Lauder et al., 2013; Yang et al., 2017). Increased inflammatory monocytes, increased TNFα+ inflammatory monocytes, and decreased neutrophils have been observed in the airway compartment in IL‐6 deficient mice during influenza infection (Dienz et al., 2012; Lauder et al., 2013). IL‐6 deficiency inhibits macrophage recruitment to the lung and reduces the phagocytic capacity of macrophages (Yang et al., 2017). Additionally, IL‐6 protects neutrophils from influenza‐mediated apoptosis (Dienz et al., 2012) and lung epithelial cells from influenza‐mediated apoptosis (Yang et al., 2017).

IL‐6 deficient mice infected with pulmonary aspergillus have increased fungal burden and inflammation on histologic examination. Increased mortality is observed in IL‐6 deficient immunosuppressed mice (Cenci et al., 2001). A trend towards decreased neutrophils in the lung has been observed in IL‐6 deficient mice following pulmonary aspergillus infection with decreased production of TNFα and increased production of MCP‐1 and IL‐17. Additionally, decreased leukocyte killing has been shown in IL‐6 deficient mice (Cenci et al., 2001). Given the beneficial effects of IL‐6 observed during both singular influenza and singular aspergillus infection, we hypothesized that IL‐6 deletion would result in defective host immunity and increased morbidity and fungal burden during IAPA. However, in this study, we demonstrate that deletion of IL‐6 during influenza infection preserves host immunity against secondary aspergillus infection. Although IL‐6 deletion caused inhibition of neutrophil accumulation in the airways and increased vascular leak during IAPA, no differences in fungal burden or overall lung inflammation were observed in the lung tissue.

Our findings reveal a complex role of IL‐6 signaling in the context of IAPA and suggest that IL‐6 deletion limits neutrophilic inflammation but enhances epithelial injury without affecting infectious burden. Decreased neutrophil accumulation in the airways of IL‐6 deficient mice does not affect fungal burden during IAPA but instead could be beneficial to the host by limiting tissue injury. However, epithelial cell damage may pose a significant unintended side effect of IL‐6 inhibitory therapies. During singular influenza infection, decreased neutrophils have been observed in the airway from Days 3–7 post influenza infection with resolution by Day 9 post‐infection (Dienz et al., 2012). Decreased phagocytic activity has been observed in both singular influenza infection and singular aspergillus infection in IL‐6 deficient mice (Cenci et al., 2001; Yang et al., 2017) and how IL‐6 signaling affects effector function during IAPA is an area that warrants additional investigation in the future. Neutrophils play a central role in the clearance of aspergillus from the lung and although reduced in the airways of the IL‐6 deficient mice, other immunologic changes are likely playing a role in fungal clearance. Increased protein content in the bronchoalveolar lavage fluid of IL‐6 deficient mice does not seem to affect lung tissue injury or fungal burden. These findings demonstrate a nuanced role of IL‐6 signaling in regulating inflammation and tissue injury during IAPA.

Cytokine and chemokine profiles in IL‐6 deficient mice demonstrated an increase in both pro‐inflammatory and anti‐inflammatory cytokines, including type I (IL‐2 and IFNγ) and type II (IL‐4 and IL‐5) cytokines in the lung. This profile is consistent with the decreased neutrophil influx in IL‐6 KO mice. During singular influenza infection, increased IFNγ, albeit not at statistically significant levels, was observed at Day 7 post‐influenza infection (Lauder et al., 2013). During singular aspergillus infection, impaired development of protective type I responses has been observed in IL‐6 deficient mice (Cenci et al., 2001). We hypothesize that the increased IFNγ levels seen in IL‐6 deficient mice during IAPA may be driven by influenza infection. Seldeslachts et al. recently reported that IFNγ is a key driver of IAPA pathogenesis, with increased IFNγ levels causing defective Th17 responses, depletion of macrophages, and inhibition of LC3‐associated phagocytosis (Seldeslachts et al., 2024) during IAPA. They observed improved fungal clearance during IAPA in IFNγ deficient mice. Our group has previously shown no difference in fungal clearance during IAPA in IFNγ deficient mice, and these observed differences may be related to varying timing and kinetics used by each group to model IAPA in mice (Tobin et al., 2020). Notably, differences in susceptibility to post‐influenza bacterial superinfection have been described when mice are challenged at different time points during the course of influenza (Rynda‐Apple et al., 2014; Shepardson et al., 2016). We also observed increased levels of RANTES and GM‐CSF in the lungs of IL‐6 deficient mice during IAPA. However, we did not observe any changes in other potent neutrophil promoting cytokines, such as KC, G‐CSF, IL‐17, IL‐1β, or TNFα. The anti‐inflammatory cytokine IL‐10 was increased in the lungs of IL‐6 deficient mice during IAPA, which may potentially affect the overall lung inflammation and contribute to the neutral changes seen in lung inflammation between wild‐type and IL‐6 deficient mice during IAPA. During singular influenza infection, increased TNFα and increased TNFα production by monocytes was observed in the airways of IL‐6 deficient mice (Lauder et al., 2013), whereas we saw no changes during IAPA.

The host ability to maintain robust antifungal immunity in the absence of IL‐6 signaling during IAPA underscores a complex role of IL‐6 signaling in post‐viral antifungal immunity. Our findings suggest that IL‐6 signaling is not required for effective clearance of fungus during IAPA and that IL‐6 signaling regulates inflammatory and injury responses in the lung. These findings are divergent from singular influenza and aspergillus infections where IL‐6 is required for both viral and fungal clearance, respectively. Taken together, our study demonstrates a complex role of IL‐6 signaling in the setting of IAPA. Findings presented here may be relevant to therapeutic modalities being used to target IL‐6 signaling, which have been used to treat patients with COVID‐19 and have been proposed to be utilized during influenza infection (Hays et al., 2022).

## 4 METHODS

### 4.1 Animals

Eight to 10‐week‐old male B6.129S2‐*Il6* ^*tm1Kopf*^/J (IL‐6 KO) mice and WT controls were purchased from The Jackson Laboratory. Mice were maintained under specific pathogen‐free conditions at the University of Pittsburgh. All the studies used age‐ and sex‐matched mice.

### 4.2 Fungal and viral infections

Influenza A/PR/8/34 H1N1 was propagated by using Madin‐Darby canine kidney (MDCK) cells. The cells were maintained in DMEM with 10% FBS (Bio‐Techne, Minneapolis, MN), penicillin (100 U/mL), and streptomycin (100 μg/mL) (Invitrogen, Waltham, MA). The cells were washed with PBS and infected with 0.001 MOI of influenza virus A/Puerto Rico/8/1934 (H1N1) in DMEM with 0.2% bovine serum albumin (Invitrogen, Waltham, MA) and 2 μg/mL of L‐tosylamido‐2‐phenyl ethyl chloromethyl ketone (TPCK) (Sigma‐Aldrich, MO). The virus‐containing supernatant was harvested after 72 h, and the viral titer was determined by standard plaque assay (provided as a generous gift by Radha Gopal, UPMC Children's Hospital of Pittsburgh, Pittsburgh, PA) (Constantinesco et al., 2024). Mice were infected with 2000 PFU of influenza A/PR/8/34 H1N1 (in 50 μL sterile PBS) from a frozen stock or control PBS by oropharyngeal aspiration under light anesthesia with isoflurane. This dose of influenza was used to achieve 15%–20% weight loss by Day 6 post‐influenza to mimic severe influenza in critically ill patients. *A. fumigatus* (American Type Culture Collection [ATCC] 42202) was maintained on potato dextrose agar for 5–7 days at 37°C. Conidia were harvested by washing the culture flask with 25 mL of sterile PBS supplemented with 0.1% Tween 20. The conidia were passed through a sterile 40‐μm nylon membrane to remove hyphal fragments and enumerated on a hemocytometer. Influenza‐infected mice were incubated for 6 days, at which time mice received 2.5 × 10^7^ conidia of *A. fumigatus* ATCC42202 inoculum or PBS control. At 48 h post‐fungal infection, all the mice were euthanized to harvest samples for further analysis. Viral burden was determined by quantitative real‐time RT‐PCR on lung RNA for viral matrix protein. For lung fungal burden analysis, the right upper lobe of each mouse was mechanically homogenized in 1 mL of sterile PBS and plated for fungal CFU counting. Burden was determined by plating 100 μL of a 1:100 dilution of lung homogenate on potato dextrose agar plates. Left lung lobes from mice were inflated with and preserved in 10% neutral‐buffered formalin solution. Tissue sections were stained with GMS, and images were collected using an automatic high‐resolution microscopic scanner by Histowiz Inc. QuPath (https://qupath.github.io), an open‐source image analysis software, was used to analyze whole slide images. QuPath has a machine learning algorithm that uses pixel classification to identify objects once a trained data set has been established. For this study, lung sections were used to train QuPath version 0.3.2 to identify GMS‐stained areas, indicating fungal conidia or hyphae. This training was then applied to all lung sections. Qupath scoring was performed by two independent operators. Lung sections were also scored by Image J to identify GMS‐stained areas, indicating fungal conidia or hyphae, performed by a third independent operator.

### 4.3 Analysis of lung inflammation

At the indicated time points, mouse lungs were lavaged with 1 mL sterile PBS for bronchoalveolar lavage cell counts and total protein levels (Bradford protein assay). The cranial lobe of the right lung was homogenized in sterile PBS by mechanical grinding. The resulting lung homogenate was used for fungal colony counting as described and cytokine analysis by Bio‐Plex Multiplex immunoassay (Bio‐Rad). RAGE levels were measured by mouse RAGE ELISA, while IgM was measured using the IgM ELISA kit.

### 4.4 Histology and analysis

Left lung lobes from mice were inflated with and preserved in 10% neutral‐buffered formalin solution. Tissue sections were stained with H&E, and images were collected using an automatic high‐resolution microscopic scanner by Histowiz Inc. QuPath (https://qupath.github.io), an open‐source image analysis software, was used to analyze whole slide images. QuPath has a machine learning algorithm that uses pixel classification to identify objects once a trained data set has been established. For this study, lung sections from vehicle and influenza‐infected mice were used to train QuPath version 0.3.2 to identify healthy parenchyma and inflamed/injured parenchyma, as well as other lung structures, like blood vessels and airways. This training was then applied to all lung sections to identify changes in healthy versus inflamed/repairing parenchyma. Lung sections were also scored by sample‐blinded histopathologic evaluation, using a severity scale of 1–4, with 4 being the greatest amount of inflammation/injury.

### 4.5 Statistics

All of the data are presented as the mean ± SEM. Significance was tested by the unpaired *t*‐test (for 2 means) or 1‐way ANOVA (for multiple data groups), followed by Tukey's post hoc test. All studies were repeated 2 times, and collected data were combined for the figures unless otherwise described. Data were analyzed using GraphPad Prism software. *p* < 0.05 was considered significant.

### 4.6 Study approval

Mouse experiments were conducted with approval from the University of Pittsburgh Institutional Animal Care and Use Committee.

## FUNDING INFORMATION

R01AI53337 (NIH) (KMR) and K2BX004886 (VA) (WB).

## ETHICS STATEMENT

All mouse experiments were approved by the University of Pittsburgh IACUC, Protocol #: 24064977.
